# Preclinical Evaluation of Novel Triphenylphosphonium Salts with Broad-Spectrum Activity

**DOI:** 10.1371/journal.pone.0013131

**Published:** 2010-10-04

**Authors:** Melissa Millard, Divya Pathania, Yumna Shabaik, Laleh Taheri, Jinxia Deng, Nouri Neamati

**Affiliations:** Department of Pharmacology and Pharmaceutical Sciences, School of Pharmacy, University of Southern California, Los Angeles, California, United States of America; Sun Yat-Sen University, China

## Abstract

**Background:**

Recently, there has been a surge of interest in developing compounds selectively targeting mitochondria for the treatment of neoplasms. The critical role of mitochondria in cellular metabolism and respiration supports this therapeutic rationale. Dysfunction in the processes of energy production and metabolism contributes to attenuation of response to pro-apoptotic stimuli and increased ROS production both of which are implicated in the initiation and progression of most human cancers.

**Methodology/Principal Findings:**

A high-throughput MTT-based screen of over 10,000 drug-like small molecules for anti-proliferative activity identified the phosphonium salts TP187, 197 and 421 as having IC_50_ concentrations in the submicromolar range. TP treatment induced cell cycle arrest independent of p53 status, as determined by analysis of DNA content in propidium iodide stained cells. In a mouse model of human breast cancer, TP-treated mice showed significantly decreased tumor growth compared to vehicle or paclitaxel treated mice. No toxicities or organ damage were observed following TP treatment. Immunohistochemical staining of tissue sections from TP187-treated tumors demonstrated a decrease in cellular proliferation and increased caspase-3 cleavage. The fluorescent properties of analog TP421 were exploited to assess subcellular uptake of TP compounds, demonstrating mitochondrial localization. Following mitochondrial uptake cells exhibited decreased oxygen consumption and concomittant increase in mitochondrial superoxide production. Proteomics analysis of results from a 600 target antibody microarray demonstrated that TP compounds significantly affected signaling pathways relevant to growth and proliferation.

**Conclusions/Significance:**

Through our continued interest in designing compounds targeting cancer-cell metabolism, the Warburg effect, and mitochondria we recently discovered a series of novel, small-molecule compounds containing a triphenylphosphine moiety that show remarkable activity in a panel of cancer cell lines as well as in a mouse model of human breast cancer. The mechanism of action includes mitochondrial localization causing decreased oxygen consumption, increased superoxide production and attenuated growth factor signaling.

## Introduction

Phosphonium salts have broad utility, with applications in chemistry, biology and pharmacology. Triphenylphosphine can easily react with alcohols, alkyl halides, and carboxylic acids giving rise to a large variety of chemical entities, which supports their wide applicability. Initially, phosphonium salts were used in preparation of phosphorus ylides, an essential component in the Wittig method of alkene synthesis.[1] As a reagent for biological research, the lipophilic, cationic properties of tetraphenylphosphonium were first utilized to demonstrate the existence of electrochemical potential across the mitochondrial membrane.[2] Charged molecules are generally unable to traverse cell membranes without the aid of transporter proteins due to the large activation energies associated with removal of associated water molecules. The distribution of charge across the large lipophilic surface of the phosphonium ion significantly lowers this energy requirement facilitating passage across lipid membranes.[3] Thus phosphonium salts accumulate in energized mitochondria due to their highly negative membrane potential. Based on this observation, the triphenylphosphonium ion has been used as a targeting moiety for delivery of agents such as spin traps, fluorescent dyes, and antioxidants to isolated mitochondria, as well as the mitochondria of intact cells and whole organisms. As pharmacological agents, certain phosphonium salts have demonstrated anti-microbial activity against gram negative and positive bacteria and the parasite *T. cruzi.*, antiglycemic properties in animal models and anti-proliferative activity in cell- and animal-based systems.[4], [5], [6], [7] As anti-cancer agents, phosphonium salts show great promise for the diagnosis and treatment of neoplasms. For reasons that are not fully understood, many solid tumors have a more negative mitochondrial membrane potential compared to their normal counterparts.[8] This trait can be exploited to allow selective delivery to tumor cells, while sparing normal cells for both treatment and imaging purposes. The first evidence of anti-proliferative activity was reported in 1978 following routine screens of synthetic intermediates.[9] In these screens isoindolylalkyl phosphonium salts showed potent anti-leukemic activity. More recently, some phosphonium salts have been reported to show anti-proliferative activity in several cancer cell lines and a xenograft model of ovarian cancer based on their ability to disrupt mitochondrial ultrastructure and alter cellular lipid content.[7], [10], [11], [12] Studies of phosphonium salts as contrast agents for diagnostic imaging [13], [14] have elucidated two key points concerning the selectivity of this class of compounds: 1) these agents are capable of preferentially accumulating within tumor cells, 2) that phosphonium cation itself does not impart cytotoxicity.

Herein we describe a series of novel compounds containing a triphenylphosphine moiety that show remarkable activity in a panel of cancer cell lines. Two of these compounds were tested in a mouse xenograft model and showed significant in vivo efficacy with no apparent toxicity. Further molecular characterization of these compounds in cell-based models suggest a mechanism of action that includes mitochondrial localization, decreased oxygen consumption, increased superoxide production and attenuated growth factor signaling.

## Results

### TP compounds are cytotoxic in a panel of human cancer cell lines

**Figure 1:**
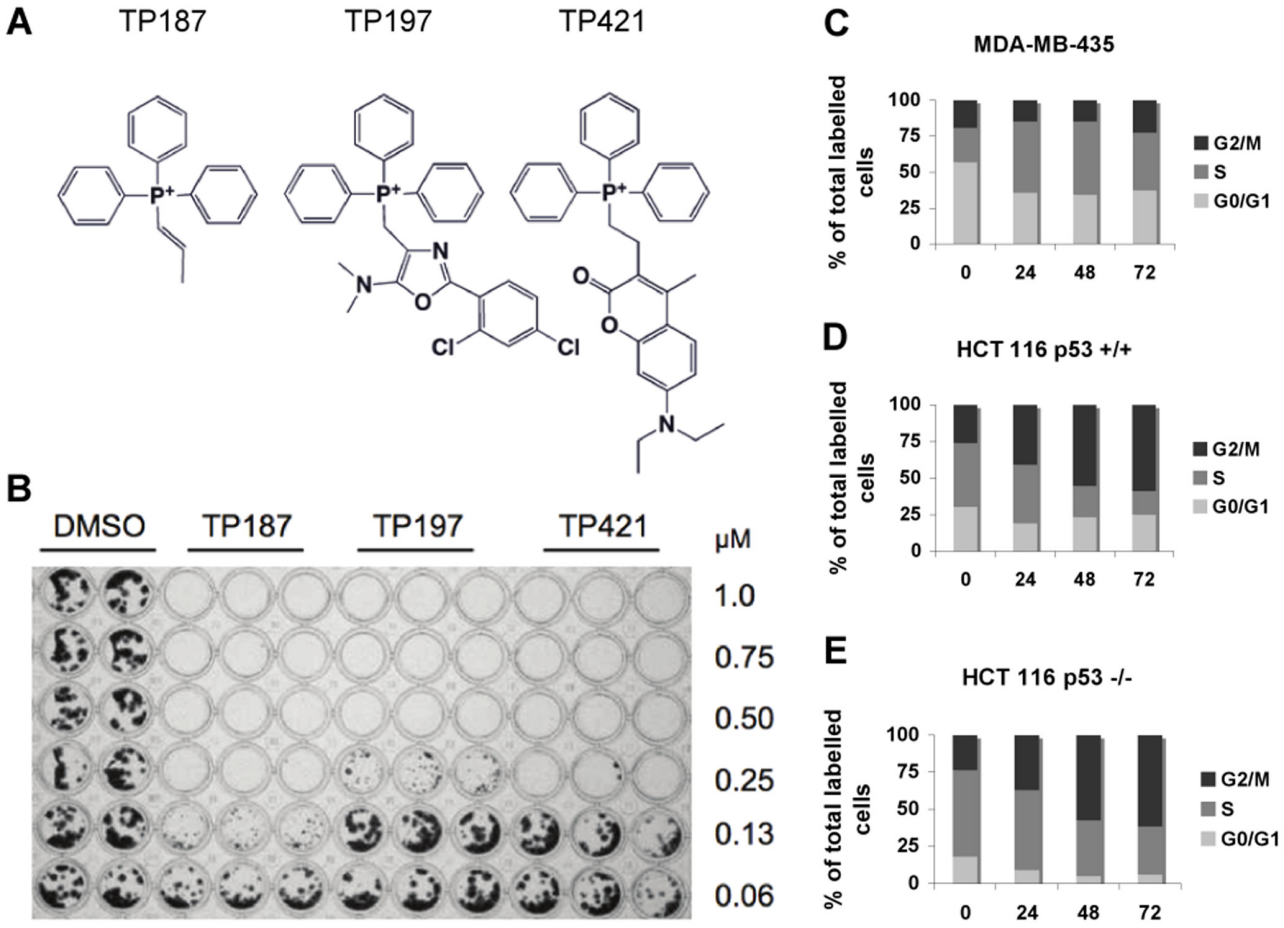
TP compounds decrease cell proliferation and induce cell cycle arrest independent of p53 status. (A) Structures of lead compounds TP187, 197 and 421. (B) Colony formation assay was performed using HCT116 p53 +/+ cells treated continuously for 7 days with TP compounds at the noted concentrations. Cells were treated in triplicate and results are representative of three separate experiments. DNA content was analyzed in propidium iodide stained MDA-MB-435 (C), HCT116 p53 +/+ (D) and HCT116 p53 −/− (E) cell lines treated for up to 72 h with 1 µM TP compounds. Light grey bars represent the G_0_/G_1_ fraction, dark grey bars represent cell in S phase and black bars correspond to cells in the G_2_/M phase of the cell cycle.

We have in place a highly diverse library of small molecules comprised of approximately 40,000 chemical entities. For the present study, a subset of 10,000 compounds predicted *in silico* to have favorable drug-like properties was selected for *in vitro* studies. Initial screening to identify active compounds was performed using a high-throughput 96-well format MTT-based cytotoxicity assay in a panel of cancer cell lines of varied origin. This screening method identified lead compounds, TP187 and TP197, having cytotoxicity values in the low micromolar range. IC_50_ values obtained in MTT assay are listed in Table 1. These results were further confirmed in colony formation assay using HCT116 p53 +/+ cells treated with increasing concentrations of TP187, 197 and the close analogue TP421 (Fig. 1A). All three TP compounds exhibited IC_50_ values in the low micromolar range across most cell lines tested in MTT (Table 1) as well as in HCT116 p53 +/+ colony assays (Fig. 1B) and were, therefore, selected for further analysis in cell- and animal-based models.

**Table 1:** Cytotoxicity of TP compounds in various cancer cell lines.

Cell line	IC_50_	
	TP187	TP197
MDA MB 435	0.8 ±0.1	0.8±0.1
MDA MB 361	12.0±0.1	0.8±0.1
MDA MB 231	2.2±0.1	1.0±0.1
PC3	1.7±0.1	0.7±0.1
DU145	11±0.1	1.2±0.1
BT474	>20±0.1	0.6±0.1
T47D	1.0±0.1	0.4±0.1
CAMA1	10±0.1	1.4±0.1
MCF7	0.8±0.1	1.3±0.1
NCI ADR RES	>20±0.1	2.0±0.1
HCT116 p53 +/+	0.4±0.1	0.4±0.1
HCT116 p53 −/−	1.3±0.1	1.3±0.1
HOP92	6.0±0.1	1.5±0.1

### TP compounds arrest cell cycle progression in human cancer cell lines

Flow cytometry was performed on ethanol-fixed propidium iodide stained tumor cell lines treated with 1 µM TP187 for 24–72 h to investigate the effect of TP compounds on cell cycle progression and DNA content. TP187 arrested cell cycle progression in all cell lines, starting at 24 h treatment. (Fig. 1C–E) MDA-MB-435, a p53 mutant cell line, arrested in the S-Phase of the cell cycle in response to treatment with TP187. (Fig. 1C) HCT116 p53 +/+ (Fig. 1D) and HCT116 p53 −/− (Fig. 1E), p53 competent and deficient cell lines, respectively, both exhibited cell cycle arrest in the G2/M phase of the cell cycle when treated with TP187. These effects on cell cycle progression were sustained through the 72 h timepoint. Based on these results, the mechanism of action was concluded to be independent of p53 status.

### TP compounds inhibit tumor growth in vivo

**Figure 2:**
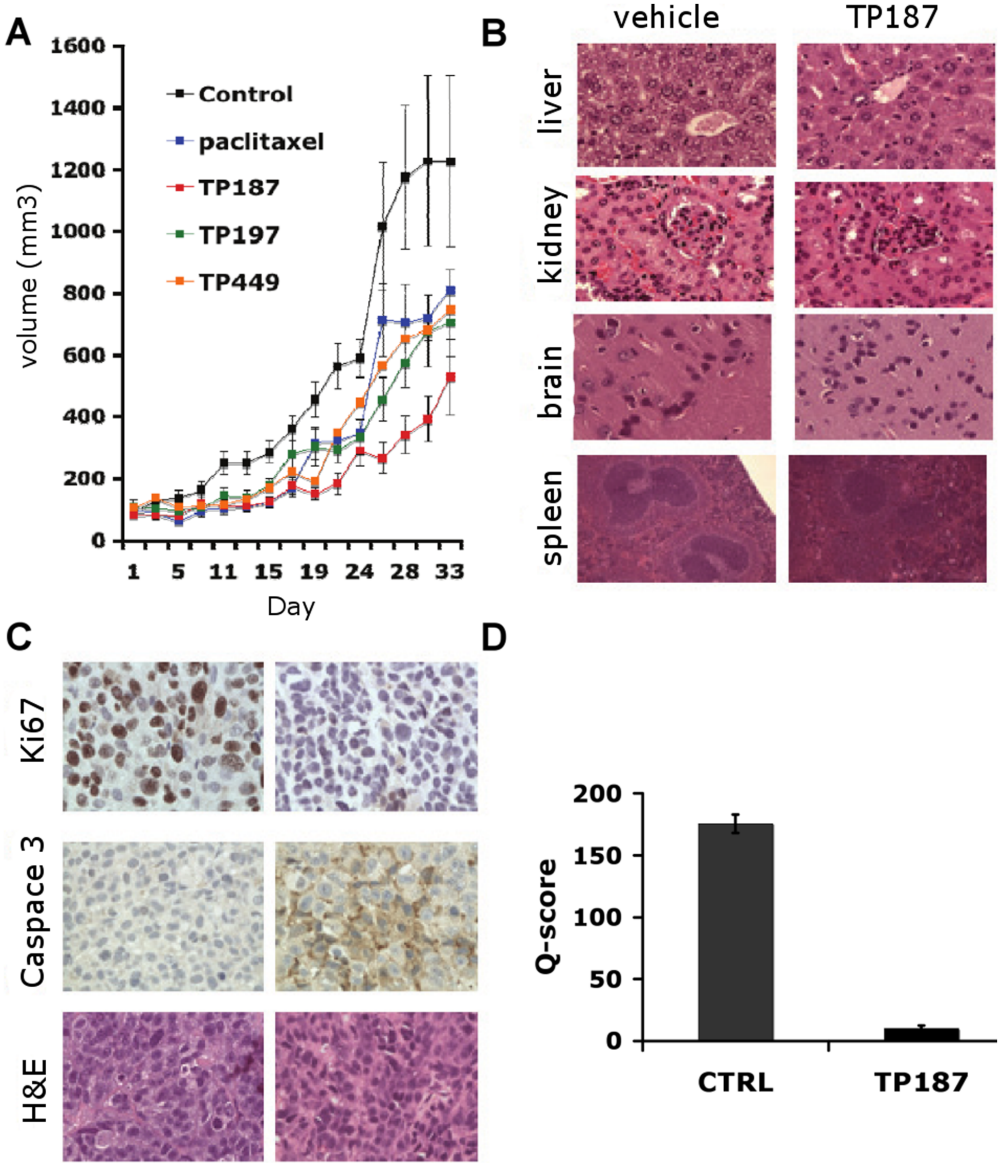
TP compounds suppress tumor growth, decrease cell proliferation and induce caspase 3 cleavage in mouse xenograft models with no systemic toxicities. (A) Graph shows average tumor volumes amongst the treatment groups over the course of the experiment. Vehicle control (green square) and TP187 (red triangle) were administered five times weekly, TP197 (open purple triangle) was administered three times weekly. Paclitaxel (closed blue circle) was administered every other day over a period of 15 days. Tumor volumes were measured three times weekly, error bars represent SEM. (B) Representative micrographs of H&E stained organ tissue collected from vehicle and TP187 treated mice. (C). Immunohistochemical staining was performed on tumor sections collected from vehicle and TP187 treated mice. Ki67 staining (upper) was significantly decreased in TP187 treated mice compared to control. Caspase 3 cleavage (middle) was increased in TP187 treated tumor sections versus vehicle treated tumors. An H&E staining of tumor sections is shown in the middle panel. Micrographs are representative of three separate experiments. (D). Graph shows the average Q-score values calculated for 10 random high power fields from the three separate mice within each treatment group. Q-score is a semi-quantitative method of tissue scoring that considers both percent and intensity of positively stained cells in a given tissue samples. Q-values were significantly decreased in TP187 treated tumor sections (p<0.001). Micrographs and Q-scores are the results of three separate experiments.

Next, we tested the TP analogues 187, 197 and 449 in a nude mouse xenograft model to determine the *in vivo* efficacy of these compounds. Treatment of MDA-MB-435 xenografts with TP compounds as single agents significantly inhibited tumor growth in both TP187 and TP197 treatment groups compared to vehicle controls. (Fig. 2A) Starting at day 13, significant differences in tumor volume were noted between TP187 and 197 treated mice compared to controls. (TP187; p<0.05, TP197; p<0.05) The suppressive effects of TP187 and 197 were maintained throughout the course of treatment (at day 33 TP187; p = 0.003 and TP197; p = 0.04). Upon conclusion of treatment at day 33, the average tumor volume of vehicle treated tumors increased by 1149%, whereas the average tumor volume in TP187 and 197 treated groups increased less than 373 and 573%, respectively. TP449 treatment also suppressed tumor growth but statistical analysis showed p values to be greater than that considered significant. (TP449; p>0.05).

### TP treated mice exhibit no sign of drug related toxicity

Mice were monitored daily by visual inspection and weighed three times per week to detect symptoms of drug related toxicity. TP-treated mice showed no outward signs of drug related toxicity such as malaise, weakness or lethargy throughout the entire 33-day treatment course. TP197 was initially administered at similar dosing but due to slight decrease in weight (<10% of body weight) within the first week, treatment was modified to dosing at 10 mg/kg, three times weekly. Soon after the dose adjustment TP197 treated mice resumed gaining weight, and by the end of the experiment weights were similar to that of the controls. Despite the reduction in number of doses of drug administered, TP197 retained its efficacy in suppression of tumor growth.

In addition to daily health checks, organ samples were collected at the time of sacrifice (day 33) to evaluate for potential drug related toxicities at the cellular level. Tissue samples derived from brain, heart, lung, liver, kidney, pancreas and spleen were harvested from mice in all treatment groups. These samples were subsequently fixed in 10% neutral buffered formalin, paraffin embedded and stained with H&E for histological analysis. Careful examination of these tissue sections demonstrated no histological abnormalities were present in tissue samples taken from TP-treated compared to control mice. Representative micrographs comparing tissue sections of liver, kidney, brain and spleen taken from control and TP187 treated mice are presented in Fig. 2B.

### TP187 decreases the number of proliferating cells and induces caspase-3 cleavage in tumor xenografts

The suppression of tumor growth in response to TP187 treatment prompted us to examine tumor sections for histological markers that could validate our *in vivo* results. Immunohistochemical staining (IHC) was performed on formalin-fixed, paraffin-embedded tumor sections collected from vehicle- and TP187-treated treated mice to evaluate the effect of treatment on cell proliferation and apoptosis. Tumor sections were processed as described in the methods section and probed using antibodies against Ki-67 and cleaved caspase-3.

Ki-67 is a cell-cycle related protein. Its presence solely in actively cycling cells makes it an ideal marker to identify the fraction of proliferating cells within a tissue sample. Ki-67 expression is increased in rapidly dividing cell populations and the degree and intensity of Ki-67 staining can be correlated with prognosis and response to treatment in many solid tumors.[15], [16] Ki67 expression was nearly absent in all TP187 treated tumor sections. A representative micrograph of the decrease in Ki-67 staining is shown in Fig. 2C, upper panel. Evaluation of Ki-67 expression using a semi-quantitative method of tissue scoring shows treatment of MDA-MB-435 xenografts with TP187 resulted in a significant decrease (p<0.001) in Ki-67 staining in comparison to vehicle treated xenografts (Fig. 2D). This result correlates well with the observed suppression of tumor growth *in vivo*.

Next we sought to determine whether our compound could induce cell death *in vivo*. Caspases are cysteine-aspartate specific proteases that, upon cleavage by upstream proteases, function in apoptotic cell death pathways. Caspase 3 is a downstream executioner caspase common to both the intrinsic and extrinsic pathways of apoptosis. Cleavage of caspase 3 is a late and irreversible event in the process of apoptosis and therefore serves as a marker for both major apoptotic pathways leading to cell death.[17] Compared to vehicle-treated, TP187-treated tumor sections showed increased cytoplasmic staining with anti-cleaved caspase 3 antibody (Fig. 2C, middle panel).

Our immunohistochemical studies showed a marked decrease in Ki-67 staining and an increase in caspase 3 cleavage in response to treatment with TP compounds. Taken together, these results suggest a mechanism, acting on effectors of cell proliferation and death pathways that together, are capable of suppressing tumor growth *in vivo*.

### TP compounds localize to the mitochondria

**Figure 3:**
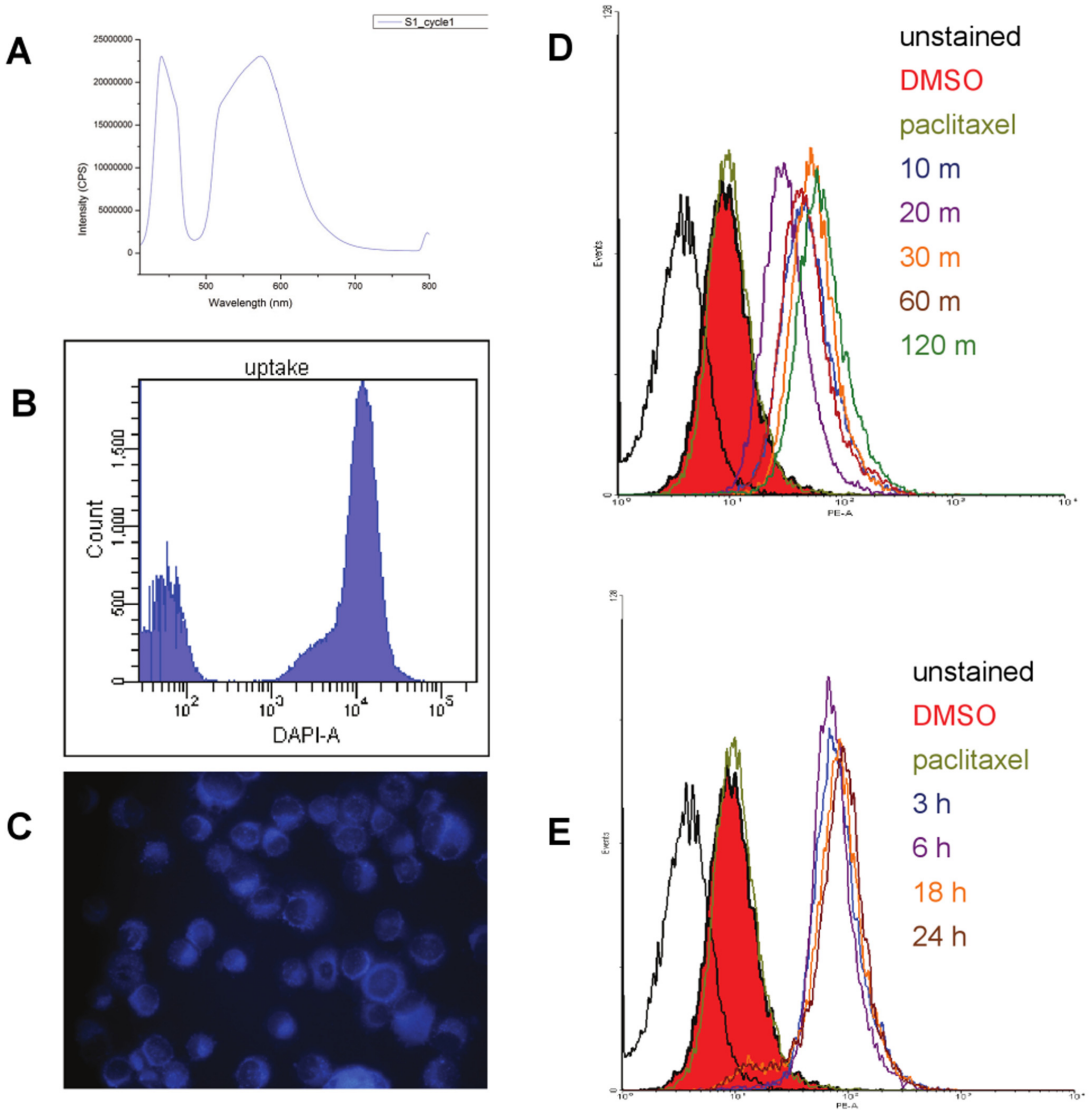
The fluorescent properties of TP421 were exploited to determine cellular uptake and localization. Upper panel, optimal excitation and emission spectra are 396 and 450 nm, respectively for TP421, as determined using steady state fluorescence spectroscopy. The graph shows the emission spectra of TP421. Middle panel, TP421 uptake can be monitored by flow cytometry. Histogram shows fluorescence intensity measured at excitation 355 nm and emission 450 nm prior to, and following treatment with TP421. Microscopy of cytospin of TP421 treated cells show subcellular localization suggestive of mitochondrial accumulation. Mitochondrial superoxide production is an early and sustained event in TP action. MDA-MB-435 cells were treated with 5 µM TP197 and superoxide production was measured at various timepoints using MitoSOX Red, a fluorogenic indicator of mitochondrial superoxide production. (D) Histogram overlays show the effect of TP treatment at early timepoints. Superoxide production increased as early as 10 minutes post-treatment (dark blue line) and maintained similar levels at 20 (purple line), 30 (orange line), 60 (brown line), and 120 (green line) minutes post-treatment. Treatment with 500 nM paclitaxel (olive line) did not increase superoxide levels above those seen in vehicle (solid red) treated cells. (E) Superoxide production is increased through later timepoints. TP-induced superoxide production was measured 3 h (dark blue line), 6 h (purple line ), 18 h (orange line), and 24 h (brown line) after TP197 treatment. Compared to vehicle (solid red) and paclitaxel (olive line) controls, TP197 caused a marked increase in superoxide production.

The favorable results obtained *in vivo* prompted us to further characterize the molecular mechanisms of TP mediated tumor suppression and identify potential targets of TP action. To this end, we chose to first investigate the subcellular localization of TP compounds in an effort to narrow the range of possible targets. The triphenylphosphonium moiety common to the TP compounds imparts a delocalized charge and lipophilic character that favors mitochondrial accumulation.[3], [18] Therefore, we tested whether our compounds could accumulate preferentially in the mitochondria by exploiting the fluorescent properties of the analog TP421. We performed fluorescence spectroscopy to determine the optimal excitation and emission wavelengths using a steady state spectrofluorimeter. TP421 has an optimal excitation wavelength of 396 nm. Excitation peaks of nearly similar intensity were observed at 450 nm and 573 nm (Fig. 3A) with 450 nm having slightly higher peak intensity.

Based on the excitation/emission spectra we were able to measure the uptake of TP421 using fluorescence-based assays. MDA-MB-435 cells were treated with 5 µM TP421 and analyzed by flow cytometry using a 355 nm UV-laser as the excitation source and optical filters capable of capturing emission in the 450 nm range. MDA-MB-435 cells treated with 5 µM TP421 displayed a sharp increase in fluorescence intensity (Fig. 3B) compared to cells treated with a comparable volume of DMSO. The intensity of fluorescence increased immediately upon addition of TP421, leveling off to a steady state with 15 minutes (data not shown) suggesting that TP uptake is rapid and likely due to the lipophilic nature and delocalized charge of the triphenylphosphonium moiety.

Next we examined the subcellular localization of TP421 using fluorescent microscopy. MDA-MB-435 cells were incubated with MitoSOX Red, following treatment with TP421. MitoSOX Red is a mitochondriotropic probe that exhibits fluorescence at excitation and emission wavelengths of 510 and 588 nm. Fluorescence microscopy of co-treated MDA-MB-435 cells revealed similar staining patterns using filters appropriate for each probe suggesting that TP421 does indeed localize to the mitochondria (Fig. 3C).

### TP compounds increase mitochondrial superoxide production

Energy production through oxidative phosphorylation is the primary function of the mitochondria. Inhibition of components of the oxidative phosphorylation machinery is known to result in the increased production of superoxide ion. [24], [25] The mitochondrial localization of TP compounds therefore, led us to investigate superoxide production as a possible mechanism of action for our compounds.

To examine the effect of TP treatment on mitochondrial superoxide production, we treated MDA-MB-435 cells with 5 µM of TP197 for varying lengths of time. At the end of treatment, cells were incubated with 5 µM of MitoSOX red and change in fluorescence intensity corresponding to production of superoxide was measured by flow cytometry. In order to rule out the possibility that our observations could be the result of non-specific ROS production in response to xenobiotic treatment, we also included MDA-MB-435 cells treated with paclitaxel.

Using this fluorogenic mitochondrial superoxide indicator, we found that TP197 treatment caused increased production of mitochondrial superoxide. MDA-MB-435 cells were treated with 5 µM TP197 and collected at various time points ranging from 10 minutes up to 24 hours. Overlays of histograms showing mean fluorescence intensity are presented in Fig. 3D. The surge in superoxide production was observed at time points as early as 10 minutes post treatment, increased to maximal intensity by 3 h post treatment and remained increased for 24 h following treatment with TP197 (Fig. 3E). Similar results were obtained measuring superoxide production in TP187 and TP421 treated MDA-MB-435 cells at 0.5 and 1, 6 and 24 h time points (data not shown). In contrast, paclitaxel treatment had no effect on the production of mitochondrial superoxide, as fluorescence intensity was unchanged compared to the vehicle control.

### TP compounds decrease the cellular rate of oxygen consumption

Based on the observed preferential accumulation of TP compounds in the mitochondria and subsequent superoxide production, we chose to examine what effect this might have on mitochondrial respiration. To this end, we measured the rates of oxygen consumption (OCR) and extracellular acidification (ECAR) in real-time using the XF 24 Extracellular Flux Analyzer. MDA-MB-435 cells were treated with either TP187, TP197, TP421 or DMEM control followed by sequential addition of oligomycin, an ATP synthase inhibitor, FCCP, an uncoupling agent and the complex I inhibitor, rotenone. OCR and ECAR were measured at short intervals over a period of 7 hours.

**Figure 4:**
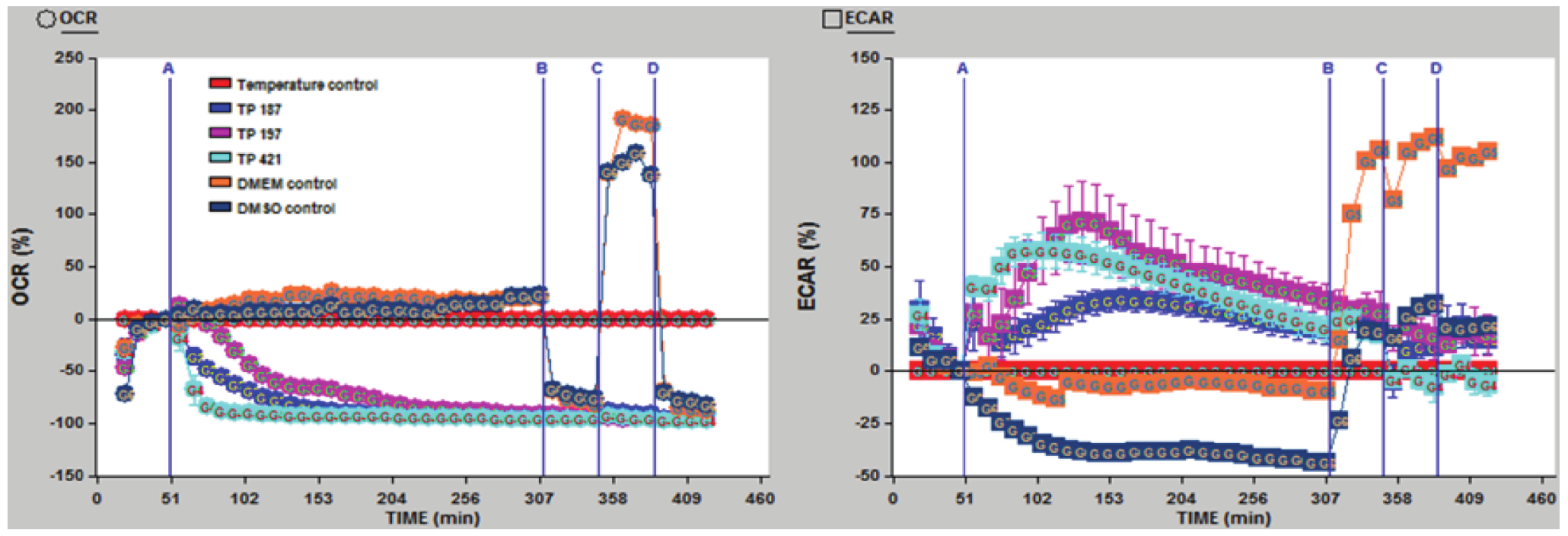
Oxygen consumption is decreased in TP treated MDA-MB-435 cells. OCR and ECAR were measured using the XF 24 Extracellular Flux Analyzer. Addition of TP compounds resulted in a decrease in OCR with a simultaneous increase in ECAR. The observed decrease in OCR was not affected by addition of inhibitors of ATP synthase, uncoupling agents or complex I inhibition. [Port A: TP compound (5 µM), Port B: Oligomycin (0.005 mg/mL), Port C: FCCP (1 µM) Port D: Rotenone (1 µM).

TP compounds decrease cellular oxygen consumption in a dose dependent manner (data not shown). Treatment of MDA-MB-435 cells with TP187, TP197 and TP421 resulted in a steady decrease in the rate of oxygen consumption (Fig. 4A). The TP-induced decrease in OCR was sustained throughout the entire 7 h time course and was unaffected by treatment with the various metabolic inhibitors. Upon addition of oligomycin, vehicle treated cells exhibited a decrease in the rate of oxygen consumption, similar to that seen with TP treatment. Meanwhile, OCR in the TP treated cells was unaffected by addition of oligomycin. Uncoupling of mitochondrial respiration from ATP synthesis by FCCP caused a large spike in the rate of oxygen consumption in vehicle treated cells, but TP treated cells did not respond at all to FCCP treatment. Addition of rotenone to vehicle treated cell completely abrogated the spike in OCR induced by FCCP, lowering OCR to levels similar to those obtained with TP treatment alone. Rotenone did not decrease the already low OCR levels in TP treated MDA-MB-435 cells. The rapid and sustained decrease in OCR suggests that TP compounds act to decrease the efficiency of oxidative phosphorylation.

The decrease in OCR in response to TP187, TP197 and TP421 was accompanied by an increase in ECAR. (Fig. 4B) Treatment with TP187 steadily increased ECAR reaching a peak rate approximately 1 hour post treatment, after this point, ECAR steadily declined yet remained above control values at all corresponding time points. Addition of TP197 caused a rapid increase in ECAR, a momentary dip in ECAR at 15 minutes post treatment and an immediate recovery leading to a peak rate at approximately 75 minutes post treatment followed by a steady decline in ECAR lasting the duration of treatment. TP421 caused a sharp increase of ECAR that reached peak rate approximately 25 minutes post treatment with a sustained downward trend. Control cells treated with oligomycin exhibited an increase in the rate of ECAR, whereas TP421 treated cells exhibited an initial decrease that quickly returned to levels seen at the TP421 treatment baseline. Oligomycin had no effect on the rate of ECAR in TP treated cells. The uncoupler, FCCP increased ECAR in control cells. TP treatment caused a momentary decrease in ECAR that was soon regained to restore levels to those seen with TP treatment alone. Rotenone caused a slight decrease in ECAR in control cells but had little affect on TP treated cells.

### Proteomics analysis of TP mechanism of action using antibody microarray

In an effort to further understand the mechanism of action of our compounds, we sought to examine their global effect on signaling proteins in MDA-MB-435 cells. To that end we employed the use of the Kinex™ KAM-1.1 Antibody Microarray. These antibody arrays are capable of detecting changes in either the phosphorylation status or the total abundance of nearly 600 well-characterized proteins representing a fraction of the total cellular proteome. Specifically, our samples were screened against 378 pan-specific (measuring total protein abundance) and 273 phospho-site-specific antibodies.

MDA-MB-435 cells were treated with either vehicle control or TP421 for 24 hours, and then collected, lysed, and sent for analysis. We selected compound TP421, because initial screening revealed it to be the most potent of the analogues in the MDA-MB-435 cell line, which was used for the proteomics experiment. All of the information obtained to date indicated that the analogues act with the same mechanism of action, though modest cell type-specific sensitivity was observed. Therefore, the results of the proteomics screen obtained using TP421 were extended to TP187 and TP197.

Of the panel of nearly 400 proteins interrogated by the chip bound antibodies, 103 proteins showed significant changes in either abundance or phosphorylation state following TP421 treatment. Those proteins identify potential signaling pathways sensitive to TP421 and likely also to its analogues, TP187 and TP197.

### TP421 and analogues affect proteins involved in key cellular functions

The proteins whose expression or post-transcriptional modification is significantly altered in response to TP treatment along with the fold change relative to control treated cells are listed in Tables 2, 3 and 4. Interestingly, many of the proteins significantly modulated are key effectors in processes or signal transduction pathways regulating cell proliferation, adhesion, intracellular survival signaling, and metabolism and gene transcription. Treatment with TP421 for 24 h significantly altered the abundance or phosphorylation of proteins involved in cell cycle entry and/or progression: CDK1, CDK7, CDC25B, p21CDK1L, cyclin D1, cyclin A1, cyclin E1, PAK3, RB (T821) and p73; focal adhesions and cytoskeletal organization: ILK1, paxillin 1, ROCK1, caldesmon, FAK (S722), Pyk2 (S579), cofilin (total and S3), vinculin (Y821), LIMK 1/2 (Y507/T508, Y504/T505); receptor tyrosine kinases: VEGFR2 (Y1054), IR (Y999), c-Kit (Y936), PDGFRa (Y742); intracellular signaling kinases and effectors: PKA Ca/b, CAMK2g, CASK/Lin2, RIP2/RICK, SIRPa1, CAMK2a (T286), PAK 1/2/3 (S144/S141/S154), PKCβ 1/2 (T500), PKCφ (T655), PKCγ (T674), PKCδ (Y313), RSK1/2 (S363/S369), and eNOS (T495); cell survival and apoptosis: PKB (S473) and JNK (T183 + Y185); cell proliferation and differentiation: MEK3b, MEK1 (S297, T385), and MEK2 (T394); regulators of transcription: STAT3, STAT6, STAT2 (Y690), and c-Jun (S73); energy sensing, glucose metabolism and transcriptional acitivation: pyruvate kinase 2, adenylate kinase 2, PCK2, mTOR (S2448), eIF4E (total & S209), eIF2a (S51). Furthermore, TP treatment increased the expression of several key intracellular phosphatases including: PP1, PP2a, PP2Cδ PP4/A'2, PP5C, PTP1C and PAC1 and decreased expression of molecular chaperones: HspBP1, Hsp60 and Grp78.

**Table 2:** Results of TP421 proteomics screen for proteins whose total abundances were decreased in response to TP421 treatment.

Protein Name	Full Protein Name	Fold Change
HspBP1	Hsp70 binding protein 1	−3.44
CK1e	Casein protein-serine kinase 1 epsilon	−3.02
CDK1 (CDC2)	Cyclin-dependent protein-serine kinase 1	−2.62
Csk	C-terminus of Src tyrosine kinase	−2.60
DAPK1	Death-associated protein kinase 1	−2.52
Cyclin D1	Cyclin D1 (PRAD1)	−2.24
CAS	Cellular apoptosis susceptibility protein (CSE1L)	−2.14
PKCa	Protein-serine kinase C alpha	−2.10
Cdc25B	Cell division cycle 25B phosphatase	−2.04
Hsp60	Heat shock 60 kDa protein 1 (chaperonin, CPN60)	−1.94
SIRPa1	Signal regulatory protein substrate of PTP1D phosphatase (SHPS1)	−1.90
ALS2CR7 (PFTAIRE2)	Amyotrophic lateral sclerosis 2 chromosomal region candidate gene protein-serine kinase 7	−1.83
Grp78	Glucose regulated protein 78	−1.79
FasL	Tumor necrosis factor ligand, member 6	−1.74
PKA Ca/b	cAMP-dependent protein-serine kinase catalytic subunit alpha/beta	−1.72
CAMK2g	Calcium/calmodulin-dependent protein-serine kinase 2 gamma	−1.59
ANKRD3	Ankyrin repeat domain protein-serine kinase 3 (RIPK4, DIK)	−1.56
CASK/Lin2	Calcium/calmodulin-dependent protein-serine kinase (Lin2 homolog)	−1.54
HO1	Heme oxygenase 1	−1.52
p21 CDKI1	Cyclin-dependent kinase inhibitor 1 (MDA6)	−1.50
ILK1	Integrin-linked protein-serine kinase 1	−1.49
PCK2	Phosphoenolpyruvate carboxykinase	−1.46
CDK1 (CDC2)	Cyclin-dependent protein-serine kinase 1	−1.45
Cyclin A	Cyclin A1	−1.42
Cdc34	Cell division cycle 34 (ubiquitin-conjugating ligase)	−1.39
AK2	Adenylate kinase 2	−1.39
p73	Tumor suppressor protein p73	−1.38
LATS1	Large tumor suppressor 1 protein-serine kinase (WARTS)	−1.37
CDC2L5 (CHED)	Cell division cycle 2-like protein-serine kinase 5	−1.35
PAK3	p21-activated serine kinase 3 (beta)	−1.34

**Table 3:** Results of TP421 proteomics screen for proteins whose total abundances were increased in response to TP421 treatment.

Protein Name	Full Protein Name	Fold Change
PAC1	Dual specificity MAP kinase protein phosphatase	1.27
STAT3	Signal transducer and activator of transcription 3 (acute phase response factor)	1.28
SPHK2	Sphingosine kinase 2	1.31
MEK3b (MAP2K3)	MAPK/ERK protein-serine kinase 3 beta isoform (MKK3 beta)	1.32
Caveolin 2	Caveolin 2	1.32
CDK7	Cyclin-dependent protein-serine kinase 7	1.35
PP1/Cg	Protein-serine phosphatase 1 - catalytic subunit - gamma isoform	1.35
PKG1	Protein-serine kinase G1 (cGMP-dependent protein kinase)	1.38
Paxillin	Paxillin 1	1.39
IKKg/NEMO	I-kappa-B kinase gamma/NF-kappa-B essential modulator(NEMO)	1.39
RIP2/RICK	Receptor-interacting serine/threonine-protein kinase 2 (RIPK2)	1.39
PKCq	Protein-serine kinase C theta	1.43
Tyro3	Tyrosine-protein kinase receptor TYRO3	1.44
DRAK2	DAP kinase-related apoptosis-inducing protein-serine kinase 2 (STK17B)	1.45
Cofilin	Cofilin 1	1.47
NT5E	Ecto-5′-nucleotidase (CD73 antigen)	1.47
eIF4E	Eukaryotic translation initiation factor 4 (mRNA cap binding protein)	1.48
PP5C	Protein-serine phosphatase 5 - catalytic subunit (PPT)	1.50
Cyclin E	Cyclin E1	1.50
Nip1	Bcl2/adenovirus E1B 19kD-interacting protein 1	1.51
ICK	Intestinal cell protein-serine kinase (MAK-related kinase (MRK))	1.52
PP4/A'2	Protein-serine phosphatase 4 - regulatory subunit (PPX/A'2)	1.52
PARP1	Poly [ADP-ribose] polymerase 1 (ADPRT)	1.54
Haspin	Haploid germ cell-specific nuclear protein-serine kinase	1.55
PKM2	Pyruvate kinase, isozymes M1/M2	1.60
TBK1	Tank-binding protein 1	1.62
IKKa	Inhibitor of NF-kappa-B protein-serine kinase alpha (CHUK)	1.66
hHR23B	UV excison repair protein RAD23 homolog B	1.74
Fyn	Fyn proto-oncogene-encoded protein-tyrosine kinase	1.74
STAT6	Signal transducer and activator of transcription 6	1.75
PCTK1 (PCTAIRE1)	PCTAIRE-1 protein-serine kinase	2.02
PP2Cd	Protein-serine phosphatase 2C - catalytic subunit - delta isoform	2.05
PTP1C	Protein-tyrosine phosphatase 1C (SHP1, SHPTP1)	2.12
IR	Insulin receptor beta chain	2.18
Aurora A (AIK)	Aurora Kinase A (serine/threonine protein kinase 6)	2.18
ROKb (ROCK1)	Rho-associated protein kinase 1	2.21
PP2A/Ca	Protein-serine phosphatase 2A - catalytic subunit alpha isoform	2.59

**Table 4:** Results of TP421 proteomics screen for proteins whose phosphorylation levels were changed in response to TP421 treatment.

Protein Name	Phosphorylation Site	Full Protein Name	Fold Change
CaMK2a	T286	Calcium/calmodulin-dependent protein-serine kinase 2 alpha	−3.02
Lck	Y191	Lymphocyte-specific protein-tyrosine kinase	−2.93
JNK	T183+Y185	Jun N-terminus protein-serine kinases (stress-activated protein kinase (SAPK)) 1/2/3	−2.90
Jun	S73	Jun proto-oncogene-encoded AP1 transcription factor	−2.68
MEK1 (MAP2K1)	S297	MAPK/ERK protein-serine kinase 1 (MKK1)	−2.61
PKA Ca/b	T197	cAMP-dependent protein-serine kinase catalytic subunit alpha/beta	−2.11
VEGFR2 (KDR)	Y1054	Vascular endothelial growth factor receptor-tyrosine kinase 2 (Flk1)	−1.96
Histone H3	T11	Histone H3.3	−1.96
RSK1/2	S363/S369	Ribosomal S6 protein-serine kinase 1/2	−1.93
IR (INSR)	Y999	Insulin receptor	−1.92
Histone H3	S28	Histone H3.3	−1.85
PAK1/2/3	S144/S141/S154	p21-activated protein-serine kinase 1/2/3	−1.84
PKCb1/2	T500	Protein-serine kinase C beta 1/2	−1.83
Jun	S73	Jun proto-oncogene-encoded AP1 transcription factor	−1.83
PKBa (Akt1)	S473	Protein-serine kinase B alpha	−1.81
Vinculin	Y821	Vinculin	−1.77
Kit	Y936	Kit/Steel factor receptor-tyrosine kinase	−1.70
MEK2 (MAP2K2)	T394	MAPK/ERK protein-serine kinase 2 (MKK2) (human)	−1.65
PKCh	T655	Protein-serine kinase C eta	−1.61
eIF2a	S51	Eukaryotic translation initiation factor 2 alpha	−1.56
MEK1 (MAP2K1)	T385	MAPK/ERK protein-serine kinase 1 (MKK1)	−1.55
LIMK1/2	Y507+T508/Y504+T505	LIM domain kinase 1/2	−1.52
eNos	T495	Nitric-oxide synthase, endothelial	−1.49
FAK	S722	Focal adhesion protein-tyrosine kinase	−1.44
Caldesmon	S789	Caldesmon	−1.42
MEK1 (MAP2K1)	S297	MAPK/ERK protein-serine kinase 1 (MKK1)	−1.35
mTOR (FRAP)	S2448	Mammalian target of rapamycin (FRAP)	1.25
STAT2	Y690	Signal transducer and activator of transcription 2	1.27
eIF4E	S209	Eukaryotic translation initiation factor 4 (mRNA cap binding protein)	1.27
Tau	S578	Microtubule-associated protein tau	1.31
PKCg	T674	Protein-serine kinase C gamma	1.35
PDGFRa	Y742	Platelet-derived growth factor receptor kinase alpha	1.52
Cofilin 1	S3	Cofilin 1	1.58
Pyk2	Y579	Protein-tyrosine kinase 2	1.58
Tau	S738	Microtubule-associated protein tau	1.75
PKCd	Y313	Protein-serine kinase C delta	2.05
Tau	S530	Microtubule-associated protein tau	2.26
Rb	T821	Retinoblastoma-associated protein 1	3.22

### Statistcal analysis of TP mechanism of action using Ingenuity Pathway Analysis

The data identifying affected proteins was further analyzed using Ingenuity software package (Ingenuity Systems Inc.) to illustrate protein-protein interaction networks, which are generated by connecting the proteins showing significant changes to TP421 treatment and relating them in biological context. In this way, the experimental data can be scrutinized for relationships, mechanisms, functions, and pathways of relevance regarding the mechanism of action of our compound.

**Figure 5:**
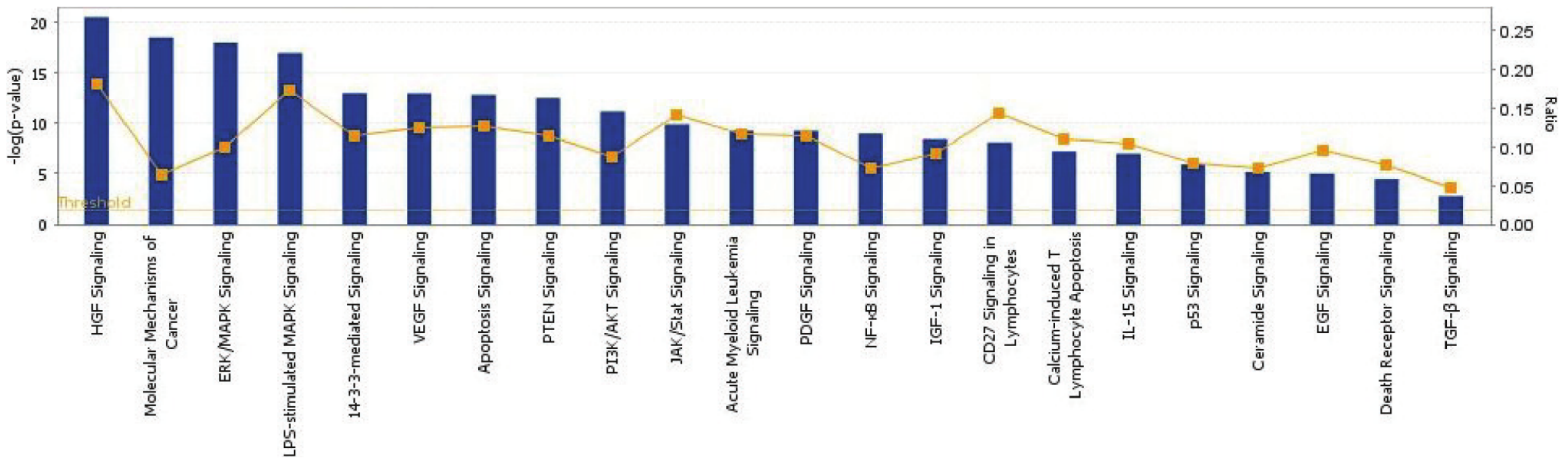
Ingenuity software analysis report of proteomics data arranged by signaling pathways in order of statistical significance. A subset of total ranked pathways is shown here.

Another global functional analysis feature displays key biological processes and pathways to visualize potentially interesting changes in tumor biology in response to TP421 treatment. In addition, the software was used to statistically rank the various pathways in order of significance (Fig. 5).

**Figure 6:**
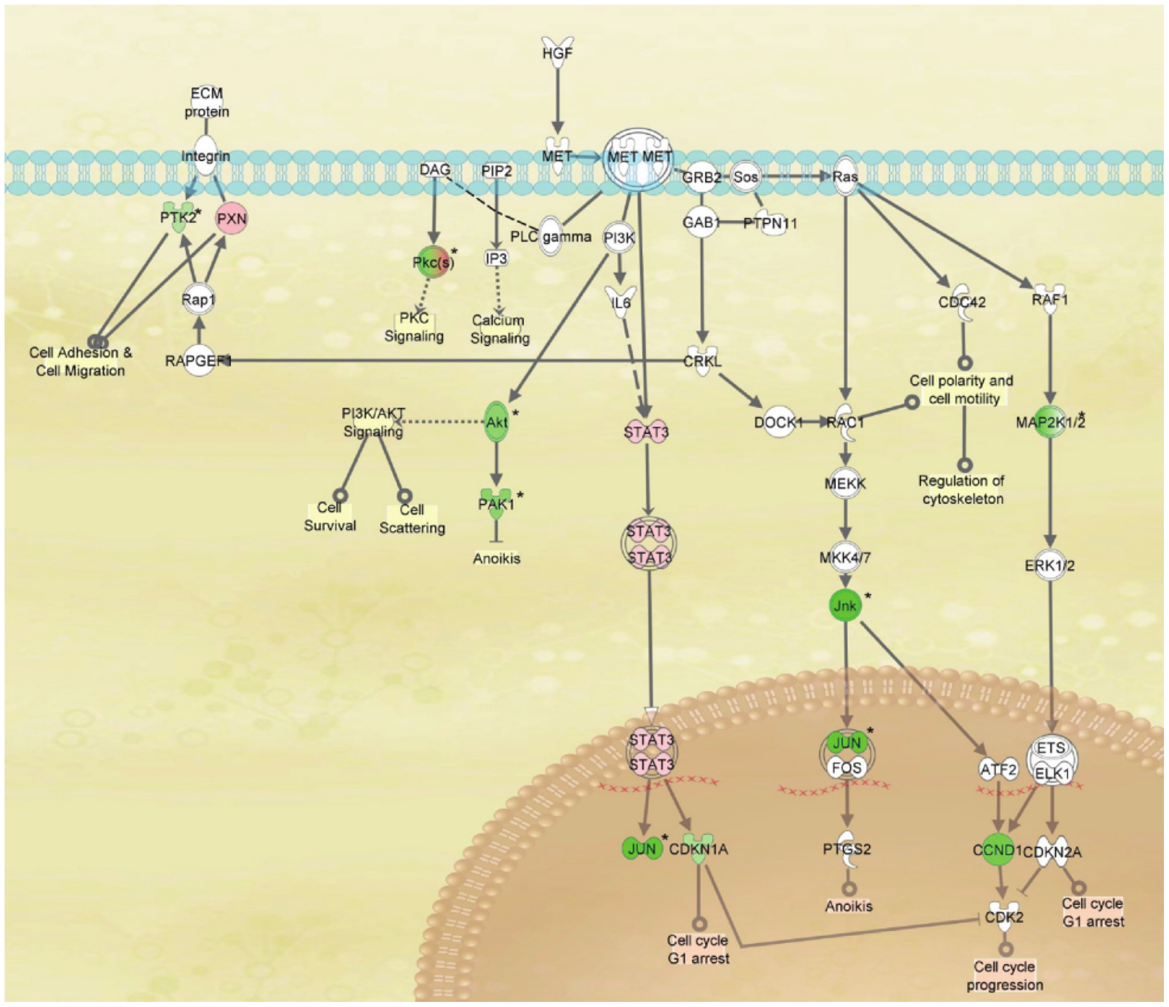
Top ranked HGF signaling pathway. Molecules in green are down-regulated in our microarray results while those in red are up-regulated. Proteins whose phosphorylation level was affected by TP421 treatment are denoted by *.

Of over 70 pathways ranked by the software, the top ranked is the Hepatocyte Growth Factor signaling pathway (Fig. 6). This pathway represents a very likely target of TP421 treatment and possibly its close analogues TP187 and TP197 as well. Several key kinases and signaling molecules in this pathway are either significantly up-regulated (seen in red) or down-regulated (seen in green) following addition of our compound to cells. This pathway is particularly intriguing because it signals to several key downstream effectors that mediate cell survival, death, adhesion and motility, and progression through the cell cycle, all of which are vital to cancer cell proliferation. For some of the proteins affected in this pathway, the total abundance was changed, while for others, specific phosphorylation sites were either increasingly phosphorylated or dephosphorylated following TP421 treatment. For the latter proteins, the effect of phosphorylation on their function is described in Table 5.

**Table 5:** Phosphorylation sites of proteins belonging to the Hepatocyte Growth Factor signaling pathway affected by TP421 treatment.

Protein Name	Phosphorylation Site	Effect on Protein Function*	Fold Change in Response to Treatment
Akt	S473	Activating phosphorylation	−1.81
FAK	S722	Unknown	−1.44
JNK	T183 + Y185	Activating phosphorylation	−2.90
c-JUN	S73	Activating phosphorylation	−2.68
MEK1	S297+T385	Induce interaction with ERK1/2 and regulate enzymatic activity	−2.61
MEK2	T394	Unknown	−1.65
PAK1/PAK2/PAK3	S144/S141/S154	May affect kinase activity	−1.84
PKC beta 1/2	T500	Unknown	−1.83
PKC delta	Y313	Promotes apoptotic effect of PKC delta	2.05
PKC gamma	T674	Unknown	1.35
PKC eta	T655	May play a role in mediating PKC eta signaling events	−1.61

*Source: www.phosphosite.org.

### Discovery of novel, active TP analogues

**Figure 7:**
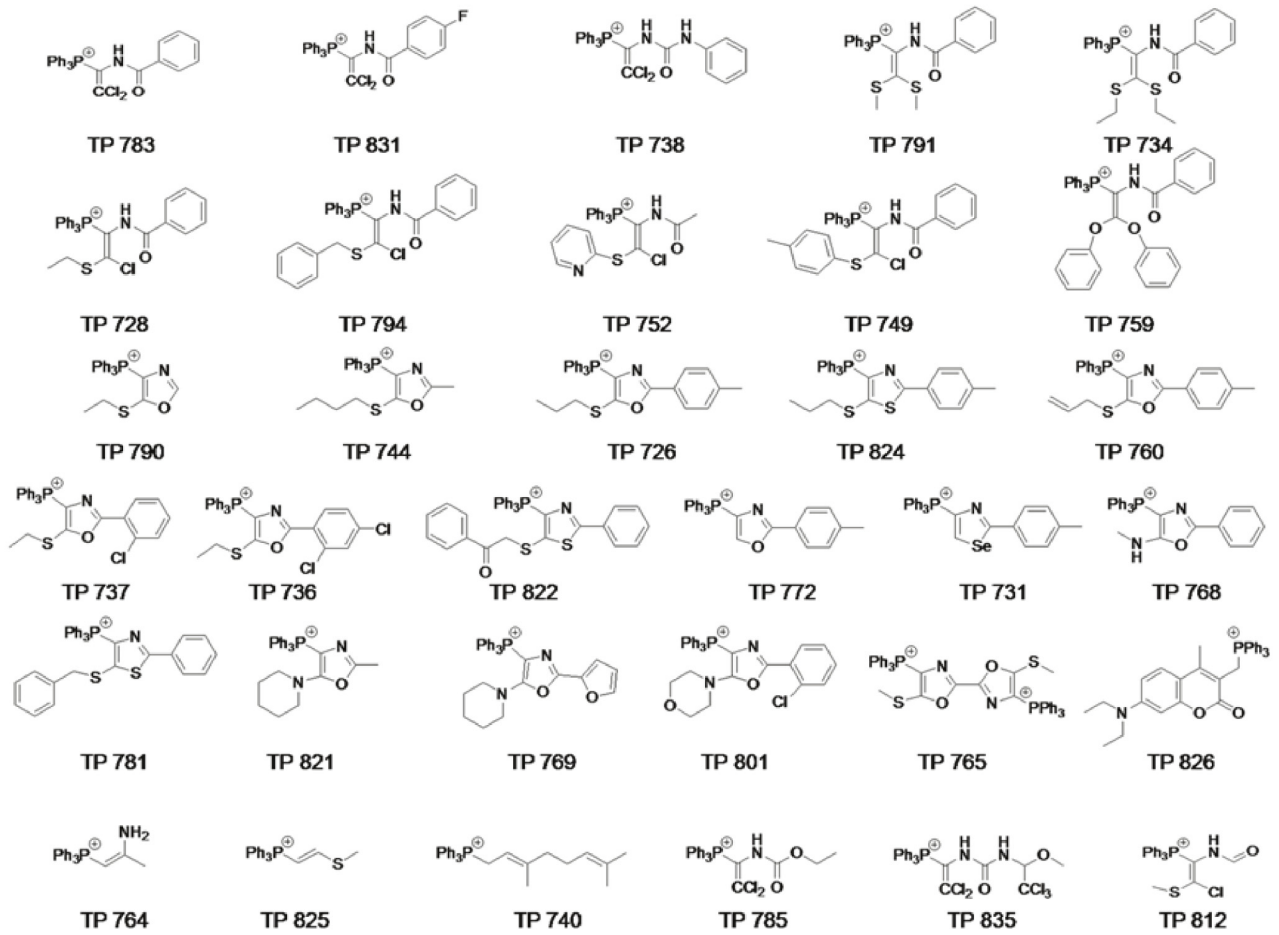
Structural analogues of TP 187, TP 197 and TP 421.

Based on the activity of TP187, TP197 and TP421, an additional 700 structural analogues containing the triphenylphosphonium moiety were designed and screened for cytotoxicity in HCT116 p53 +/+, a colon carcinoma cell line. Compounds displaying more than 50% cytotoxicity at 10 µM were selected for further testing in a panel of five cancer cell lines: MDA-MB-435, MCF-7, PC3, HCT116 p53+/+ and HCT 116 p53-/-. The compounds having IC_50_ values ≤5 µM in MTT assay are displayed in Fig. 7 and results are reported in Table 6. The cytotoxicity of these compounds was subsequently tested by colony formation assay. With the exception of a few compounds, a good correlation was observed between the results obtained in the two assays. (Table 6) These outcomes indicate that the presence of a TP moiety alone is not sufficient for cytotoxicity. Although a number of close analogues of TP187, TP197 and TP421 were screened, we were unable to identify a coherent structure activity relationship between analogues due to similar levels of activity between analogues.

**Table 6:** IC_50_ of structural analogues of TP 187, TP 197 and TP 421 in a panel of cancer cell lines.

S.No.	Compound	Molecular weight (G)	IC_50_ (µM)									
			MCF-7		MDA-MB435		PC3		HCT116 p53+/+		HCT116 p53-/-	
			MTT	CFA	MTT	CFA	MTT	CFA	MTT	CFA	MTT	CFA
1	TP 783	477.34	2.8	2.2	1.6	3.8	2.8	3	2	1.4	2.7	1.8
2	TP 831	495.33	2.8	3.5	3.4	4.1	3	1.1	2.8	1.6	2.7	3.8
3	TP 738	492.36	3.2	2.8	3.8	2.2	2.8	1	2.8	2.8	3.2	3.1
4	TP 791	500.63	4	3	4	2.9	3.8	3	3.5	5	5.8	>10
5	TP 734	528.69	4.2	2.4	4.2	10	3.4	4	5	>10	6	>10
6	TP 728	503.01	3.8	1.1	3.4	2.8	3.7	3.1	3.1	2.9	3.1	2.3
7	TP 794	565.08	3	2.4	2.8	3.5	3	3.1	2.8	3.2	2.6	3
8	TP 752	489.98	5	3.2	8.4	>10	5	4	4	6	5	5
9	TP 749	565.08	2.6	>10	2.5	>10	2.2	8.5	3.1	>10	3.1	4
10	TP 759	592.64	3.1	0.8	3.8	4	3	2.9	1.8	3	3.1	2.8
11	TP 790	390.46	0.8	3.2	0.78	4.1	3	3.2	2.8	6	3.2	3
12	TP 744	432.54	0.65	0.3	2.5	2	3	3.4	1.5	3.1	1.8	3.9
13	TP 726	494.61	0.5	0.15	0.6	2.1	0.5	3.2	0.4	2.4	0.58	2.8
14	TP 824	510.67	0.3	0.38	0.6	1.5	0.42	0.22	0.3	0.6	0.4	0.48
15	TP 760	492.59	0.5	0.28	0.98	0.18	3	0.31	0.31	0.6	0.42	0.78
16	TP 737	501.00	0.5	0.31	1	1.1	0.58	0.6	1.8	3.2	2.5	3
17	TP 736	535.44	5	2.8	5	4	4	3.4	3.8	3	4	3
18	TP 822	572.70	1	2.8	1.5	6	2.8	2	3.5	2.8	2.9	3.2
19	TP 772	420.46	0.65	0.7	5	3.2	1.6	0.6	0.9	2.8	3.1	3
20	TP 731	483.42	0.45	0.2	0.5	0.35	0.4	0.28	0.25	0.28	0.35	0.32
21	TP 768	435.48	0.45	1.1	0.5	3.2	3	0.8	0.99	3.1	2.1	3.8
22	TP 781	544.69	3.2	2.2	4	3.1	5	2.4	1.8	4	3.5	3.8
23	TP 821	427.50	0.55	1.1	1.8	7	3.8	1.8	2.4	3.1	3.8	2.2
24	TP 769	479.53	0.7	0.8	0.5	0.45	1	0.39	0.4	2.8	1.9	2.2
25	TP 801	525.98	3.2	2.4	6	4.1	4	2.8	2.8	3	2.8	3
26	TP 765	750.85	2.4	1.1	>10	3	4.1	2.8	2	3	4	3.8
27	TP 826	506.59	0.58	0.9	0.6	4	0.8	0.25	0.56	2	1.2	1.8
28	TP 764	318.37	7	6	6	>10	8	>10	9	>10	>10	>10
29	TP 825	335.42	1.2	4.5	4	5.1	5	2.2	3.5	3.1	3.9	3
30	TP 740	399.53	2	0.9	2.1	2	1.2	0.7	0.72	2.6	2.8	3
31	TP 785	445.30	2.7	2.2	3.4	3.8	2.8	3.5	2.8	2.8	3	2.3
32	TP 835	577.67	3	3	3.6	3	2	0.7	2.5	1.2	2.2	3.2
33	TP 812	412.89	3.1	1.5	3.2	3.5	3.1	>10	2.8	2.9	3.8	3.7

## Discussion

The tumor microenvironment is characterized by alterations in oxygen and nutrient content based, in part, on the inadequacies of tumor vasculature. The tortuous, disorganized tumor vascular bed produces hypoxic, nutrient poor regions within growing tumors. Survival in these sub-optimal conditions requires tumor cells invoke adaptive strategies to circumvent nutrient deprivation and hypoxia. Alterations in energy metabolic pathways and the adaptive responses to hypoxia and nutrient deprivation are emerging as hallmarks of cellular transformation. [19] Midway into the 20^th^ century, Warburg postulated that even with adequate oxygenation, cancer cells rely on glycolytic metabolism as an adaptive mechanism to compensate for dysfunction at the level of mitochondrial respiratory chain.[20] More recent knowledge demonstrates results to the contrary in some tumor types suggesting the Warburg effect may not apply to all cancers. Poor tumor cell perfusion limits glucose supply considerably making glycolysis energetically unsustainable. Measured oxygen levels in hypoxic tumor tissues are higher than that at which respiratory dysfunction is thought to occur, precluding hypoxia as a cause of mitochondrial dysfunction.[21] Through examining the contributions of each metabolic pathway to cell proliferation it has been observed that oxidative phosphorylation supplies a large portion of the ATP produced in many tumor types, particularly in the absence of glucose. Furthermore, cell-based studies demonstrate an increase in cancer cells' oxidative phosphorylation capacity and oxygen affinity upon prolonged culture in glucose free media and the requirement of functional mitochondria to activate survival mechanisms such as the unfolded protein response (UPR) pathway under glucose starved conditions.[22], [23] All of which are not observed in non-transformed cell lines. Tumors exhibit additional metabolic changes, including de novo lipid and nucleotide biosynthesis and glutamine dependent anaplerosis. These alterations allow for growth under adverse conditions, generation of substrates for glycosylation reactions, and supply of precursors for biosynthetic reactions [24], [25]. Often, TCA cycle intermediates are co-opted as precursors for the biosynthetic reactions. Citrate derived from the TCA cycle is utilized as a precursor for fatty acid synthesis. Oxaloacteate and α-ketoglutarate provide nonessential amino acids required for protein and nucleotide synthesis. To compensate, cancer cells have higher uptake of glutamine for replenishing TCA cycle intermediates through glutaminolysis.[26] Taken together, these evidence suggest that drugs targeting the mitochondrial respiratory capacity should have a profound effect on tumor growth while sparing normal cells.

Herein we have identified a series novel triphenylphosphine based compounds exhibiting potent anti-tumor activity. Our preliminary screening of TP analogues 187, 197 and 421 demonstrated cytotoxicity in a panel of cancer cell lines of varied origin and cytogenetic attributes, inhibition of colony formation at submicromolar concentrations and cell cycle arrest independent of p53 competence. TP analogues 187 and 197 significantly suppressed the growth and proliferation of MDA-MB-435 tumors in a mouse xenograft at clinically achievable dosing. Daily health monitoring and post-mortem histology revealed no detectable systemic toxicities or drug related tissue injury. Collectively, these results demonstrate the potential clinical utility of TP compounds in the treatment of a wide range of cancer types.

Using the fluorescent analog TP421 we were able to confirm rapid uptake and mitochondrial localization as is expected for compounds containing the triphenylphosphine moiety. Early events in TP induced apoptosis included a decrease in the rate of oxygen consumption, a concomitant increase in superoxide production and transient increase in glycolytic capacity. TP treatment of MDA-MB-435 cells caused an immediate and sustained decrease in oxygen consumption that was unaffected by the inhibition of ATP synthase, uncoupling of mitochondrial respiration and blockade of complex I function. The observed decrease in OCR was accompanied by a temporal increase in superoxide production beginning as early as ten minutes post treatment and continuing for up to 24h and a transient increase in the rate of extracellular acidification indicative of glycolysis. It is likely these early events contribute significantly, if not wholly, to the TP mechanism of action.

**Figure 8:**
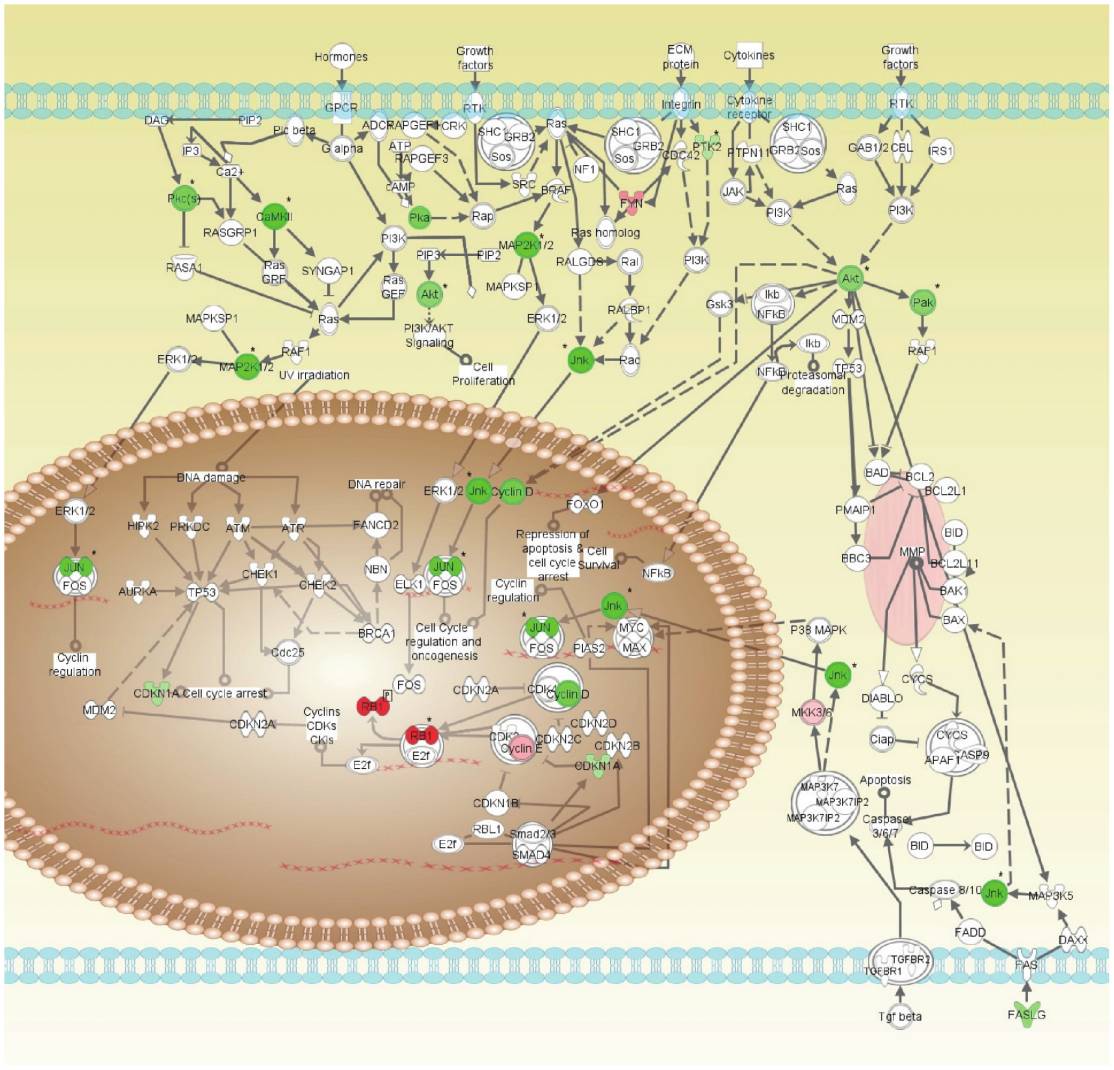
Proposed mechanism of action. TP compounds reduce mitochondrial oxygen consumption, and increase superoxide leading to the formation of ROS species that cause attenuation of signaling pathways involved in cancer cell growth and proliferation. Using the IPA platform, proteins exhibiting significantly up- or down- regulated expression or phosphorylation (denoted by *) were examined in the context of signaling pathways involved in cancer cell survival mediated by growth factors, cytokines, cell adhesion and hormones. TP-treatment impinged on key signaling molecules in the MAPK (mitogen activated protein kinase), JNK (c-jun N-terminal kinase), PI3K/Akt/mTOR (phosphotidylinostiol-3-kinase/Akt/mammalian target of Rapamycin), and CAMK (calcium-calmodulin kinase) pathways leading to suppression of cyclin D1 expression and c-jun phosphorylation coupled with an increase in Rb phosphorylation.

To our knowledge, this is the first paper to evaluate the cellular response to triphenylphosphonium compounds at the proteomic level, and thus provides insight on the retrograde mitochondria-to-proteome response. A mechanism for the late action of TP analogues can be related to the significant alterations observed in signal transduction cascades relevant to cancer cell biology after 24 hours treatment. (Fig. 8) In response to TP421, MDA-MB-435 cells showed a marked down-regulation of key signal transducers governing cellular responses to growth factors, cytokines, hormones, and adhesion to extracellular matrix that ultimately impinged upon the expression of cyclins D1 and E, retinoblastoma protein phosphorylation and phopshorylation of the c-jun transcription factor. In addition to the pathways depicted above, significant decreases in expression or activation state were noted for proteins involved in or related to the regulation of cell cycle progression, protein folding and chaperoning, intracellular calcium signaling, mitogenic signaling, and cell adhesion. TP treatment also significantly increased the abundance and activation state of numerous protein serine phosphatases, activators of NFkB, PKC isoforms and the members of the STAT family of transcription regulators. These alterations in protein abundance and function, due to their ability to modulate cell fate, are also likely contributors to the later phase mechanisms of TP action.

Although the data reported here does not elucidate the exact relation between the earlier and later responses and the degree to which each contributes to the outcome of TP response, it is possible to predict a generalized mechanism of action based on the probable interplay of temporal events.

Superoxide produced in the mitochondria in response to inhibition of oxygen consumption can be converted to hydrogen peroxide, hydroxyl radical or in the presence of nitric oxide, peroxynitrite.[27], [28], [29], [30] These ROS derivatives can diffuse or be transported out of the mitochondria to enter the cytosolic and nuclear compartments. ROS can react with thiols of cysteine and methionine residues of proteins, in both the cytosol and nucleus, causing either intra- or extracellular disulfide linkages. Disulfide linkages modify protein structure, which can directly impact function thorough alterations in protein activity, protein/protein associations and sub-cellular localization. In the nucleus, ROS can also act to inhibit transcription factors by altering their redox status while higher concentrations of ROS have been shown to induce oxidative DNA damage.[31], [32]

The cellular response to ROS production is dependent on the cellular redox buffering potential as well degree and duration of ROS production. Abrupt, intense ROS over production may overwhelm cellular antioxidant responses causing irreversible oxidative damage to cellular proteins, lipids and DNA leading to oxidative stress and ultimately cell necrosis. Lower levels of ROS production, on the other hand, produce milder oxidative imbalances that via redox mediated regulation of signal transduction result in programmed responses such as cell proliferation, senescence and apoptosis.[32], [33]

Based on this knowledge, it is plausible that TP compounds, through selective inhibition of tumor cell oxygen consumption and subsequent superoxide generation in the mitochondria, contribute to sustained, low levels of ROS production. The sustained levels of ROS eventually create an imbalance in the cellular redox status that causes modulation in the function of proteins involved in cell cycle regulation, growth factor signaling and DNA transcription resulting in the global protein expression profile observed in the Kinexus data. Dyfunction in these molecular effectors impinges on pathways critical to cancer cell survival, proliferation and interaction with the extracellular environment leading to the induction of apoptosis.

Full elucidation of the mechanism of TP action is beyond the scope of this report but, with these analyses, we demonstrate that TP analogues impact multiple pathways relevant to cancer cell survival, growth, cell cycle progression, cell adhesion and motility and apoptosis, many of which have demonstrated sensitivity to intracellular redox status. Taken together the results of our studies involving TP compounds suggest a unique mechanism of action that includes inhibition of oxygen consumption leading to sustained increases in free radical production which alter redox sensitive cell signaling pathways to inhibit growth factor mediated signaling, promote cell cycle arrest and induce apoptosis.

## Materials and Methods

### Cell culture

MDA-MB-435 breast, MCF7 breast cancer and PC3 prostate cancer cell lines were purchased from the American Type Cell Culture (Manassas, VA). HCT116 p53+/+ and HCT116 p53-/- cells were kindly provided by Dr. Bert Vogelstein (Johns Hopkins Medical Institutions, Baltimore, MD). Cell lines were maintained in the appropriate growth media (DMEM for MDA-MB-435, MCF7 and PC3, RPMI for the HCT116 cell lines) containing 10% heat-inactivated fetal bovine serum and supplemented with 2 mM L-gutamine at 37°C in a humidified atmosphere of 5% CO2. For subculture and experiments cells were washed with 1x PBS, detached using 0.025% Trypsin-EDTA (Cellgro, Mediatech, Mannassas, VA), collected in growth media and centrifuged. All experiments were performed in growth media using sub-confluent cells in the exponential growth phase. For use in tissue culture experiments, compounds were prepared at 10 mM concentration in sterile dimethylsulfoxide (DMSO) (EMD Chemicals, Gibbstown, NJ) and stored at −20°C when not in use.

### Cytotoxicity assay

Cytotoxicity was assessed by a 3-(4,5-dimethylthiazol-2-yl)-2,5-diphenyltetrazolium bromide (MTT) assay as previously described.[34] Cells were seeded in 96-well tissue culture treated dishes and allowed to adhere overnight. Cells were subsequently treated with a continuous exposure to drugs for 72 hours. An MTT solution was added to each well to give a final concentration of 0.3 mg/mL MTT. Cells were incubated with MTT for 3–4 hours at 37°C. After removal of the supernatant, DMSO was added and the absorbance was read at 570 nm. All assays were done in triplicate. The IC_50_ was then determined for each drug from a plot of log drug concentration versus percentage of cell kill.

### Colony formation assay

Colony formation assay was performed as previously described [35] to further assess drug toxicity. To this end, cells were seeded in 96 well tissue culture dishes at a density of 200 cells per well in growth media and allowed to adhere overnight. Cells were subsequently treated with varying concentrations of compound for 24 h. Following treatment, monolayers were washed with 1x PBS and incubated in growth media for a period of 7–10 days, allowing sufficient time for colonies to form in control wells. To visualize the extent of colony formation, cells were fixed and stained in a 2% solution of crystal violet containing 1% glutaraldehyde. Excess stain was removed through multiple washes in distilled water and allowed to air dry. Stained plates were imaged using Quantity One software running on the VersaDoc imaging platform. (BioRAD). Quantification by measurement of optical density at 570 nm was performed after solublization in a 2% solution of sodium dodecyl sulfate accompanied by 2 h shaking on a platform rocker.

### Cell cycle analysis

Cells were seeded in 100 mm tissue culture dishes at a density of 1×10^6^ cells/plate in growth media and allowed to adhere overnight. The following day cells were treated with IC_80_ concentrations of TP compounds or DMSO alone as vehicle control for 24–72 h. Upon completion of treatment, cells were detached with trypsin and both media and cells were collected by centrifugation. Cells were washed and resuspended in 1x PBS prior to fixation in ethanol overnight at −20°C. Fixed cells were treated with 10 µg/mL RNase A (Sigma-Aldrich, St. Louis, MO) and stained in a 50 µg/mL solution of propidium iodide (Sigma-Aldrich, St. Louis, MO). DNA content was determined by flow cytometry using the BD LSR II (BD Biosciences, San Jose, CA) equipped with a 488 nM Sapphire™ argon-ion laser and PE emission detector.

### Xenograft studies

1.5×10^6^ MDA-MB435 cells were implanted subcutaneously into the rear flank of 6-week old female nu/nu mice (Charles River Laboratories, Wilmington, MA). When tumor volumes reached 100 mm^3^, mice were separated into one of six treatment groups consisting of four to eight mice per group. Treatments were administered by intraperitoneal injection in a 50 µL suspension of 5% DMSO/95% peanut oil (v/v). Group 1 (n = 8) received vehicle control of DMSO/peanut oil. Group 2 (n = 4) received 10 mg/kg body weight paclitaxel every other day for a total of seven doses. Group 3 (n = 8) received 10 mg/kg body weight TP187, five times weekly. Group 4 (n = 8) received 10 mg/kg body weight TP197 every other day. Group 5 (n = 8) received 10 mg/kg body weight TP449 five times weekly. Tumor volumes and weights were measured three times weekly. Tumors were measured using calipers. Tumor volume was calculated using the following equation: V =  d^2^ × D/2 , where d = the width or smaller measure and D =  the length or larger measure. Data collected was plotted and analyzed to determine average tumor volumes and weights, SEM values, and p-values using Microsoft Excel. Health checks were performed daily. Mice exhibiting toxicities or excessive tumor burden (> 2.0 cm^3^) were sacrificed using CO_2_ gas, necropsies were performed and tumor samples and organs were harvested and fixed in 10% neutral buffered formalin prior to processing for histological analysis. Upon completion of the experiment remaining mice were sacrificed and following necropsy, the organs harvested, fixed and processed for histological analysis. The animal studies were approved by the USC Animal Care and Use Committee under protocol numbers 10766 and 11458. Animal care and manipulation were in agreement with the USC institutional guidelines, which were in accordance with the Guidelines for the Care and Use of Laboratory Animals.

### Tissue handling

Surgically excised tissues or organs were washed in 1x PBS to remove blood and bodily fluids prior to fixation in 10% neutral buffered formalin. Samples were fixed for 24–48 hour after which time the organs were stored in 1x PBS until ready to process for analysis. Fixed samples were placed in cassettes and processed for histological analysis using the Microm STP 120 spin tissue processor (Richard Allen Scientific, Kalamazoo, MI). At the completion of the processing, tissues/organs were embedded in molds containing hot paraffin and allowed to solidify on the Microm EC 350-2 refrigerated cooling tray. Paraffin blocks were cooled on ice and 4-micron sections were cut using a Microm HM 310 microtome (Microm International GmBH, Walldorf, Germany). Paraffin embedded sections were floated in a Thermo Scientific Tissue Flotation bath filled with water heated to 44°C prior to mounting on pre-cleaned, positively-charged glass slides (Richard Allen Scientific, Kalamazoo, MI). Slides were placed upright and allowed sufficient time to air dry.

### Hematoxylin and eosin staining

H&E staining was performed using Thermo Scientific Shandon Varistain Gemini automated stainer (Richard Allen Scientific, Kalamazoo, MI). The Gemini stainer was programmed as follows. Slides were deparaffinized in the heater block for 20 minutes. The program then continued with incubation of slides in 3 changes of Clear-Rite 3 for three minutes followed by two changes of FLEX100 for one minute each. The slides were then incubated in FLEX 95 for one minute before a running water wash. After the water step, slides were stained with Hematoxylin 7211(Richard Allen Scientific, Kalamazoo, MI) for two minutes, thirty seconds followed by a one minute running water wash. Next, the slides were incubated one minute with Clarifier 2 (Richard Allen Scientific, Kalamazoo, MI) to remove background hematoxylin staining. Clarifier 2 treatment was followed with a one-minute running water wash prior to a one-minute incubation with bluing reagent (Richard Allen Scientific, Kalamazoo, MI). After the bluing reagent, the slides were washed one minute in running water and then incubated for thirty seconds in FLEX 95. The slides were then stained with Eosin Y (Richard Allen Scientific, Kalamazoo, MI ). Eosin Y staining was followed with three consecutive one minute washes in 100% FLEX and finally three consecutive changes of Clear-rite 3 (Richard Allen Scientific, Kalamazoo, MI). The slides were then removed from the Gemini stainer and coverslipped using 1–2 drops of mounting media (Richard Allen Scientific,) and air dried several hours. Specimens were examined by light microscopy. Slides were visualized using a Ziess axioscope light microscope equipped with 10 x eyepiece and 5, 20, 40 and 100 x objectives. Light micrographs were obtained using Moticam 2300 microscope camera (Motic in North America, Richmond, BC, Canada).

### Immunoperoxidase staining of formalin-fixed paraffin-embedded tissue sections

Tissue sections four microns thick were mounted on pre-cleaned positively charged glass slides. Tissue sections were deparaffinized using three changes of xylenes for five minutes each. Sections were hydrated, first in two washes of 100% ethanol for 10 minutes each, then two washes in 95% ethanol for 10 minutes each followed by immersion in double distilled water (ddH_2_O) for one minute. Antigen retrieval was performed by boiling slides for ten minutes in 10 mM sodium citrate pH 6.0. Immunohistochemical staining was performed using the UltraVision ONE detection system (Thermo Scientific, Fremont, CA) according to the manufacturer's protocol. MDM2 (clone SMP14) was obtained from Biosource, Invitrogen, (Carlsbad, CA). p53 antibody (clone DO-1) was obtained from Santa Cruz Antibodies (Santa Cruz, CA) and used at dilutions of 1∶500. Ki67 antibody (clone SP6) was obtained from Thermo Scientific (Fremont, CA) and was used at a dilution of 1∶400. Anti-cleaved Caspase 3 antibody was purchased from Cell Signaling (Danvers, MA) and was used at a 1∶400 dilution. IgG isotype controls for rabbit and mouse were purchased from Santa Cruz antibodies and used at dilutions of 1∶400 and 1∶500 as negative controls in all staining procedures. Immunolabelled sections were counterstained for 10 seconds with hematoxylin 7211 (Richard Allen Scientific, Kalamazoo, MI) and rinsed in ddH_2_O three to four times to remove excess stain. Tissue sections were then dehydrated through two ten-second washes in 95% and 100% FLEX alcohol (Richard Allen Scientific, Kalamazoo, MI), followed by three five-second changes of Clear-rite 3. Excess clearite was blotted and slides were mounted using clarion mounting medium (Sigma-Aldrich, St. Louis, MO) and glass coverslips. Slides were air-dried overnight prior to microscopy.

### Q-Score method for quantification of nuclear immunohistochemical staining

Q-score is a semi-quantitative method of tissue scoring using the formula Q =  P x I, where P is equal to the percentage of positively staining cells and I is equal to the intensity at which each positve cell stains. I or intensity, is given a value between 1 and 3, representing low, medium or high staining intensity. To score Ki-67 expression in control verses TP187 treated tumor sections, three tumor sections from each treatment were examined. For each, at least 10 random fields were photographed and P and I values were calculated for each individual field. The average Q-score and the corresponding SEM were calculated in this way for each treatment.

### Statistical analysis

Statistical analysis was performed for tumor volumes and Q-scoring of immunohistochemical staining in Microsoft Excel using the Student's t-test, assuming unequal variances. P-values less than 0.05, obtained by this method were considered to be significant.

### Flow cytometric analysis of TP421 uptake

The fluorescent properties of TP421 were exploited to investigate the cellular uptake of TP compounds. PC3 prostate cancer cell lines were seeded at a density of 5.0×10^5^ cells/dish in 33 mm tissue culture-treated dishes and allowed to adhere overnight in 2 mL growth media. The following day cells were collected by trypsinization, washed and resuspended in 500 µL 1x PBS. Three minutes prior to, and immediately following addition of 10 µM TP421 or DMSO, as vehicle control, fluorescence versus time was recorded for emission wavelengths between 425–475 nM in response to excitation with the 355 nM UV solid state laser of the BD LSR II flow cytometer.

### Fluorescence spectroscopy

Fluorescence spectroscopy was performed on TP compounds and MitoSOX to determine optimal excitation and emission wavelengths using the Fluorolog 3, steady state spectrofluorimeter (Horiba Scientific, Edison, NJ). Spectroscopy was performed using 5–10 µM compound dissolved in dimethylsulfoxide (DMSO) (EMD Chemicals, Gibbstown, NJ) resuspended in 2 mL ddH_2_O. Readings were corrected to remove background fluorescence corresponding to DMSO and ddH_2_O.

### Flow cytometric analysis of ROS formation

MDA-MB-435 cancer cells were treated with 2 µM TP compounds for various time periods prior to incubation with 5 µM MitoSOX Red Mitochondrial Superoxide Indicator (Invitrogen, Carlsbad, CA) at 37°C for 10 minutes. Cells were trypsinized, washed three times with Hank's Balanced Salt Solution (HBSS) to remove residual MitoSOX before resuspending in 750 µL 1x PBS. Fluorescence intensity corresponding to oxidation of MitoSOX Red by superoxide radicals was recorded for emission wavelengths between 562–588 nM in response to excitation with the 488 nM Sapphire™ argon-ion laser of the BD LSR II flow cytometer.

### Bioenergetics studies

Bioenergetics studies were conducted using an XF 24 Extracellular Flux Analyzer (Seahorse Bioscience, Billerica, MA). The assays were performed in a disposable sensor cartridge containing 24 pairs of fluorescent biosensors coupled to a fiberoptic waveguide, and a 24 well XF24 cell culture microplate.

To prepare the biosensor cartridge, the biosensors were hydrated by adding 1 mL of XF24 calibrant solution (Seahorse Bioscience, Billerica, MA) in each well of the XF24 biosensor cartridge. The cartridge was then incubated overnight at 37°C without an external CO_2_ source. Concurrently, MDA-MB-435 cells were seeded at a density of 120,000 cells per well in a 100 µL culture medium in each of 20 wells of the XF 24 cell microplate leaving A1, B4, C3 and D6 control wells blank. The cells were allowed 4 h to adhere prior to the addition of 150 µL of culture media to each well. The plate was subsequently incubated overnight at 37°C in the presence of 5% CO_2_.

At the onset of bioenergetics measurements, the assay medium was warmed to 37°C, and the pH was adjusted to 7.4. Culture media was removed from the XF24 cell microplate, and the wells were rinsed with 1 mL assay medium. Next, 600 µL of the assay medium was added to each well, and the XF24 cell microplate was incubated at 37°C for 1 h without CO_2_ supplementation. Dilutions of the stock compounds were prepared in assay media. A volume of 60 µL reagent was added the injection ports of the biosensor cartridge, and were maintained at 37°C without CO_2_ supplementation. Calibration and assay measurements were performed at 37°C. The data generated reflects measurements taken over a treatment period of seven hours.

### Bioenergetics assay medium

The bioenergetics assay medium was prepared by dissolving DMEM base (8.3 g/L, Sigma, St. Louis, MO) in 500 mL distilled water. 1.85 g of sodium chloride (Sigma, St. Louis, MO) was dissolved separately in 500 mL of distilled water. Solutions of sodium chloride and DMEM base were then combined and 20 mL of this combined solution was replaced with 10 mL of 100 x GlutaMax-1 (Gibco), and 10 mL of 100 mM sodium pyruvate (Sigma-Aldrich, St. Louis, MO). The media was then warmed to 37°C. The pH of the media was adjusted to 7.4 using 5 M sodium hydroxide (Sigma, St. Louis, MO). Finally, the media was sterilized by filtration and stored at 4°C for future use at which time the temperature and pH of the media were again adjusted to 37°C and 7.4, respectively on the day of the assay.

### Kinexus antibody microarray

MDA-MB-435 cells were treated with 1 µM of TP421 for 24 h. Upon completion of treatment, cells were washed in ice-cold PBS to remove residual medium, then lysed in 200 µL of lysis buffer (20 mM MOPS, pH 7.0, 2 mM EGTA, 5 mM EDTA, 30 mM sodium fluoride, 60 mM β-glycerophosphate, pH 7.2, 20 mM sodium pyrophosphate, 1 mM sodium orthovanadate, 1 mM phenylmethylsulfonylfluoride, 3 mM benzamidine, 5 µM pepstatin A, 10 µM leupeptin, 1% Triton X-100, 1 mM dithiothreitol) and collected. The cell lysates were sonicated on ice, four times for 10 s each pausing for 15 s intervals between pulses, to rupture the cells and shear DNA. After sonication, the homogenates were cleared by centrifugation at 90,000× g for 30 min at 4°C. The supernatants were transferred to a clean microcentrifuge tube and the protein concentrations were measured using the BCA protein assay. Whole cell lysates in a final volume of 250 µL were submitted to Kinexus for a 628-antibody microarray analysis using the Kinex™ KAM-1.1 Antibody Microarray (Kinexus Bioinformatics Corp).

### Ingenuity Pathway Analysis

Potential intracellular signaling pathways or molecules affected by TP421 treatment were identified using the Ingenuity Pathway Analysis (IPA) software to analyze the Kinexus™ antibody microarray results. The significantly up-regulated or down-regulated pan-specific or phopsho-proteins with their Swiss-Prot accession numbers and the ratio changes were uploaded as an Excel spreadsheet file to the IPA server. TP421-mediated signaling pathways were analyzed by core analysis.

## References

[1] Wittig G (1980). From Diyls to Ylides to My Idyll.. Science.

[2] Grinius L, Jasaitis A, Kadziauskas Y, Liberman E, Skulachev V (1970). Conversion of biomembrane-produced energy into electric form. I. Submitochondrial particles.. Biochim Biophys Acta.

[3] Murphy M (2008). Targeting lipophilic cations to mitochondria.. Biochim Biophys Acta.

[4] Bergeron KL, Murphy EL, Majofodun O, Munoz LD, Williams JC (2009). Arylphosphonium salts interact with DNA to modulate cytotoxicity.. Mutation Research-Genetic Toxicology and Environmental Mutagenesis.

[5] Kinnamon K, Steck E, Hanson W, Chapman WJ (1977). In search of anti-Trypanosoma cruzi drugs: new leads from a mouse model.. J Med Chem.

[6] Blank B, DiTullio N, Deviney L, Roberts J, Saunders H (1975). Synthesis and hypoglycemic activity of phenacyltriphenylphosphoranes and phosphonium salts.. J Med Chem.

[7] Rideout D, Calogeropoulou T, Jaworski J, Dagnino RJ, McCarthy M (1989). Phosphonium salts exhibiting selective anti-carcinoma activity in vitro.. Anticancer Drug Des.

[8] Modica-Napolitano J, Aprille J (2001). Delocalized lipophilic cations selectively target the mitochondria of carcinoma cells.. Adv Drug Deliv Rev.

[9] Dubois R, Lin C, Beisler J (1978). Synthesis and antitumor properties of some isoindolylalkylphosphonium salts.. J Med Chem.

[10] Rideout D, Bustamante A, Patel J (1994). Mechanism of inhibition of FaDu hypopharyngeal carcinoma cell growth by tetraphenylphosphonium chloride.. Int J Cancer.

[11] Manetta A, Gamboa G, Nasseri A, Podnos Y, Emma D (1996). Novel phosphonium salts display in vitro and in vivo cytotoxic activity against human ovarian cancer cell lines.. Gynecol Oncol.

[12] Cooper WA, Bartier WA, Rideout DC, Delikatny EJ (2001). 1H NMR visible lipids are induced by phosphonium salts and 5-fluorouracil in human breast cancer cells.. Magn Reson Med.

[13] Wang J, Yang C, Kim Y, Sreerama S, Cao Q (2007). 64Cu-Labeled triphenylphosphonium and triphenylarsonium cations as highly tumor-selective imaging agents.. J Med Chem.

[14] Kim Y, Yang C, Wang J, Wang L, Li Z (2008). Effects of targeting moiety, linker, bifunctional chelator, and molecular charge on biological properties of 64Cu-labeled triphenylphosphonium cations.. J Med Chem.

[15] Isola J, Helin H, Helle M, Kallioniemi O (1990). Evaluation of cell proliferation in breast carcinoma. Comparison of Ki-67 immunohistochemical study, DNA flow cytometric analysis, and mitotic count.. Cancer.

[16] Veronese S, Gambacorta M, Gottardi O, Scanzi F, Ferrari M (1993). Proliferation index as a prognostic marker in breast cancer.. Cancer.

[17] Li J, Yuan J (2008). Caspases in apoptosis and beyond.. Oncogene.

[18] Ross M, Kelso G, Blaikie F, James A, CochemÈ H (2005). Lipophilic triphenylphosphonium cations as tools in mitochondrial bioenergetics and free radical biology.. Biochemistry (Mosc).

[19] Pathania D, Millard M, Neamati N (2009). Opportunities in discovery and delivery of anticancer drugs targeting mitochondria and cancer cell metabolism.. Adv Drug Deliv Rev.

[20] Warburg O (1956). On the origin of cancer.. Science.

[21] Moreno-Sánchez R, Rodríguez-Enríquez S, Marín-Hernández A, Saavedra E (2007). Energy metabolism in tumor cells.. The FEBS Journal.

[22] Smolková K, Bellance N, Scandurra F, Génot E, Gnaiger E (2010). Mitochondrial bioenergetic adaptations of breast cancer cells to aglycemia and hypoxia.. J Bioenerg Biomembr.

[23] Haga N, Saito S, Tsukumo Y, Sakurai J, Furuno A (2010). Mitochondria regulate the unfolded protein response leading to cancer cell survival under glucose deprivation conditions.. Cancer Sci.

[24] DeBerardinis RalphJ, Lum JulianJ, Georgia Hatzivassiliou, Thompson C (2008). The Biology of Cancer: Metabolic Reprogramming Fuels Cell Growth and Proliferation.. Cell Metabolism.

[25] Gatenby RA, Gawlinski ET, Gmitro AF, Kaylor B, Gillies R (2006). Acid-mediated tumor invasion: a multidisciplinary study.. Cancer Cell Research.

[26] DeBerardinis RJ, Sayed N, Ditsworth D, Thompson CB (2008). Brick by brick: metabolism and tumor cell growth.. Current Opinion in Genetics and Development.

[27] Deby C, Goutier R (1990). New perspectives on the biochemistry of superoxide anion and the efficiency of superoxide dismutases.. Biochem Pharmacol.

[28] Fridovich I (1978). The biology of oxygen radicals.. Science.

[29] Steinbeck M, Khan A, Karnovsky M (1993). Extracellular production of singlet oxygen by stimulated macrophages quantified using 9,10-diphenylanthracene and perylene in a polystyrene film.. J Biol Chem.

[30] Chance B, Williams G (1956). The respiratory chain and oxidative phosphorylation.. Adv Enzymol Relat Subj Biochem.

[31] Cromlish J, Roeder R (1989). Human transcription factor IIIC (TFIIIC). Purification, polypeptide structure, and the involvement of thiol groups in specific DNA binding.. J Biol Chem.

[32] Holmgren A (1985). Thioredoxin.. Annu Rev Biochem.

[33] Filomeni G, Rotilio G, Ciriolo M (2005). Disulfide relays and phosphorylative cascades: partners in redox-mediated signaling pathways.. Cell Death Differ.

[34] Carmichael J, DeGraff W, Gazdar A, Minna J, Mitchell J (1987). Evaluation of a tetrazolium-based semiautomated colorimetric assay: assessment of chemosensitivity testing.. Cancer Res.

[35] Munshi A, Hobbs M, Meyn R (2005). Clonogenic cell survival assay.. Methods Mol Med.

